# Synchrotron XRF Imaging Reveals Manganese Accumulation in the Golgi and Post‐Synapses of Neurons and Enhanced Uptake in Astrocytes

**DOI:** 10.1002/advs.202520364

**Published:** 2026-04-09

**Authors:** Ines Kelkoul, Aiyarin Kittilukkana, Luis C. C. Huarte, Hiram Castillo‐Michel, Murielle Salome, Stéphane Roudeau, Pauline Belzanne, Matthieu Sainlos, Noémie Pied, Monica Fernandez‐Monreal, Daniel Choquet, Richard Ortega, Asuncion Carmona

**Affiliations:** ^1^ University of Bordeaux CNRS LP2I Bordeaux UMR 5797 Chemical Imaging and Speciation in Neuroscience Gradignan France; ^2^ ESRF, Beamline ID21 The European Synchrotron Grenoble France; ^3^ University of Bordeaux CNRS Interdisciplinary Institute for Neuroscience Bordeaux France; ^4^ University of Bordeaux CNRS INSERM Bordeaux Imaging Center Bordeaux France

**Keywords:** manganese, metal contamination, neurotoxicology, single cell, synchrotron imaging

## Abstract

Manganese is an essential trace metal, but excessive exposure causes neurotoxicity, including parkinsonian syndromes, cognitive deficits, and may contribute to neurodegenerative diseases. Worldwide, tens of millions are exposed to elevated manganese in drinking water, exceeding World Health Organization guidelines. Despite its importance for public health, the cellular and subcellular mechanisms of manganese neurotoxicity remain poorly understood, particularly its distribution among brain cells and intracellular targets. We examined manganese accumulation in primary rat hippocampal neurons and astrocytes using a correlative imaging approach combining cryo‐fluorescence light microscopy and synchrotron X‐ray fluorescence imaging to map and quantify manganese at subcellular resolution. Manganese preferentially accumulated in the Golgi apparatus of neurons and astrocytes. In neurons, it was also present at the postsynaptic density, suggesting a role in synaptic vulnerability. Quantitative analysis showed that astrocytes accumulated about three times more manganese than neurons. Neuronal manganese uptake was reduced when neurons were co‐cultured with astrocytes, indicating a potential protective or buffering function of astrocytes. These findings identify critical cellular and subcellular targets of manganese, highlighting the Golgi apparatus as a central site in manganese neurotoxicity. This work advances understanding of cell type–specific responses to manganese exposure and may guide the development of targeted neuroprotective strategies.

## Graphical abstract

Despite widespread exposure, the mechanisms of manganese neurotoxicity remain poorly understood. Using correlative cryogenic fluorescence and synchrotron X‐ray fluorescence imaging techniques, we show that the Golgi apparatus is the primary accumulation site for manganese in both neurons and astrocytes, and that manganese targets the neuronal postsynaptic density. Astrocytes accumulate more manganese than neurons, suggesting a buffering neuroprotective role.

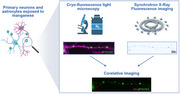

## 1 Introduction

Manganese (Mn) is an essential element required by the body to perform its physiological functions properly. It is involved in the metabolism of many proteins and lipids, as well as the detoxification of reactive species. It also plays a role in energy metabolism and glucose regulation [1, 2]. In the central nervous system, Mn acts as a cofactor for specific enzymes, such as glutamine synthetase. However, Mn is also a toxic element that primarily affects the central nervous system (for full reviews see [3, 4, 5]). The balance between essentiality and toxicity is narrow [3, 6], and overexposure to Mn can lead to various neurological disorders, including motor dysfunction and cognitive impairment [3, 4, 5]. Chronic exposure to high levels of Mn is associated with the development of ‘manganism’, a parkinsonian syndrome whose symptoms resemble those of Parkinson's disease and include cognitive impairment [7, 8]. Mn may also increase the risk of developing idiopathic Parkinson's disease [9]. Genetically, mutations in the solute carrier proteins SLC30A10 and SLC39A14, which are involved in Mn transport, have been identified in individuals with familial parkinsonism, leading to severe motor symptoms and mild cognitive impairment [4]. Furthermore, various epidemiological studies support the notion that prolonged exposure to Mn can result in multiple cognitive impairments in children [10, 11, 12, 13, 14, 15]. Tens of millions of people worldwide are at risk of being exposed to excessive levels of Mn through their drinking water [16]. Recently, ambient Mn exposure was associated with cortical brain atrophy in the general adult population [17].

Following overexposure, Mn accumulates in the basal ganglia, particularly in the globus pallidus, but is also observed in other brain regions, such as the choroid plexus, substantia nigra, striatum, frontal cortex, cerebellum, and hippocampus [3, 18, 19, 20, 21, 22, 23, 24]. At the cellular level, studies show that mitochondrial function and microglia‐mediated neuroinflammation are affected after Mn exposure [25]. Mn also induces the dysregulation of autophagy and exocytosis, as well as causing oxidative stress, endoplasmic reticulum stress, and apoptosis, and interfering with neurotransmitter metabolism [26, 27, 28]. Mn affects mitochondrial morphology and function in both astrocytes [29] and neurons [30]. In neurons, Mn exposure significantly affects dendritic length, spine density, and the number of dendritic endings [31, 32]. Mn targets cell signaling pathways and interferes with neurotransmitter systems, such as the dopaminergic, cholinergic, glutamatergic, and GABAergic systems, through multiple mechanisms, including direct interaction with neurotransmitter receptors [28, 33]. However, the subcellular distribution of Mn in neurons still needs to be identified.

Astrocytes play an active and crucial role in Mn homeostasis in the central nervous system, mediating both protection and neurotoxicity [34, 35]. It is assumed that astrocytes accumulate Mn^2+^ ions through DMT1 (divalent metal transporter 1) and/or by Mn^3+^ binding to the transferrin receptor. In astrocytes, Mn is required for the activity of glutamine synthetase, which catalyzes the conversion of glutamate and ammonium ions into glutamine. Glutamine synthetase contains eight Mn atoms per octamer and accounts for 80% of the total Mn in the brain [36]. Astrocytes may accumulate Mn preferentially to neurons, 10 to 50 times more [34, 35], although quantitative data, such as exact intracellular concentrations in each cell type under identical conditions, have not been reported. When present in excess, Mn can disrupt the glutamate/GABA‐glutamine shuttle, leading to neuronal damage and contributing to the neurodegenerative process [37].

We used synchrotron X‐ray fluorescence (SXRF) quantitative imaging to compare the distribution of Mn in single primary rat hippocampal neurons and astrocytes in the same co‐culture, which had been exposed to a sub‐cytotoxic concentration of Mn. We used hippocampal neurons and astrocytes as well‐characterized models to study fundamental mechanisms of Mn neurotoxicity. This model is relevant to environmental and occupational exposure scenarios, such as chronic ingestion or inhalation, conditions under which Mn has been detected in the hippocampus [18, 19, 20, 21, 22, 23]. Additionally, using a correlative protocol for imaging metals and proteins [38, 39], we have elucidated the subcellular distribution of Mn in neurons and astrocytes. This could be achieved using fluorescent markers of the Golgi apparatus and of the postsynaptic density, a protein dense structure attached to the postsynaptic membrane essential for maintaining synaptic strength and plasticity. This information is key to evaluating the potential protective role of astrocytes, understanding neurotoxicity mechanisms, highlighting subcellular targets, as well as proposing detoxification strategies.

## 2 Materials and Methods

### 2.1 Culture of Primary Neurons and Astrocytes for SXRF Imaging

In order to enable SXRF analyses, neurons must be cultured on substrates compatible with this technique. To this end, we employed silicon nitride membranes (Si_3_N_4_) with a thickness of 500 nm (SiRN‐5.0‐200‐1.5‐500, Silson Ltd.). The protocol published previously [38, 40, 41, 42] was followed, as adapted from Kaech & Banker's method for culturing hippocampal neurons from embryonic rats [43]. In our protocol, Si_3_N_4_ membranes were sterilized by immersion in absolute ethanol for 2 h. Any remaining ethanol was removed by washing the membranes twice with sterile, ultra‐trace elemental analysis‐grade water (Fisher Scientific, France), after which the membranes were dried for 2 h under a laminar flow hood. To allow cell adhesion, the membranes were coated with 1 mg/mL poly‐*L*‐lysine (PLL, P2636, Sigma‐Aldrich) in 0.1 m borate buffer (B6768, Sigma‐Aldrich) for 2.5 h at 37°C. The membranes were then washed twice with sterile ultra‐trace elemental analysis grade water and covered with neurobasal culture medium (Gibco, 12349015) to prevent PLL drying and crystal formation.

Here, we describe the specifics of each type of labelling and cell seeding in our study. Primary hippocampal neurons and astrocytes from 18‐day‐old (E18) Sprague–Dawley rat embryos were seeded and cultured in three different ways according to the experimental objectives, known as the ‘neuron condition’, ‘co‐culture condition’, and ‘neuron PSD (post‐synaptic density) condition’, as explained below. Experimental procedures were in accordance with the European Guide for the Care of Animals used for Experimental and Other Scientific Purposes and the animal care guidelines of the Research Ethics Committee of the University of Bordeaux.

For the culture condition called ‘neuron condition’, dissociated neurons were seeded onto the PLL‐coated Si_3_N_4_ membranes at a homogeneous density of 14 000 cells/cm^2^ using equilibrated Neurobasal medium supplemented with 0.5 mm GluTmAX (Gibco, 35050038) and B‐27 Plus (Gibco, A3582801). This completed culture medium is called NBB27. Two hours after seeding (minimum time to ensure cell adhesion), the membranes were gently transferred to a Petri dish (60 mm diameter) containing an astrocyte feeder layer with 5 mL NBB27 medium and returned to the incubator. At 3 days in vitro (DIV3), 2 µm AraC (cytosine α‐*D‐*arabinofuranoside hydrochloride, Sigma, C1768) was added to the medium to stop astrocyte cell proliferation in the Petri dish. Neurons were cultured until DIV15, and once per week, 1 mL of equilibrated NBB27 was added to compensate for evaporation. In this ‘neuron condition’, only neurons were present on the Si_3_N_4_ membranes, with astrocytes remaining on the Petri dish.

For the culture condition called ‘co‐culture condition’, primary rat hippocampal neurons and astrocytes were seeded together on Si_3_N_4_ membranes. For this purpose, the PLL‐coated Si_3_N_4_ membranes were placed in a coated Petri dish (60 mm diameter) containing 5 mL of NBB27 and 1.5% horse serum. In this case, 14 000 cells/cm^2^ were homogeneously seeded onto the Petri dish containing the membranes and immediately returned to the incubator to allow astrocytes and neurons to develop. This ensured both cell types would adhere and grow on the Si_3_N_4_ membranes. At DIV3, the medium was replaced with equilibrated NBB27 without serum. Neurons and astrocytes were cultured until DIV15, and once per week, 1 mL of equilibrated NBB27 was added to compensate for evaporation. In this condition, the Si_3_N_4_ membranes contained neurons and astrocytes.

In order to image Mn in dendritic spines for the ‘neuron PSD condition’, neurons must be cultured more than 15 days in close contact with an astrocyte feeder layer. To identify the postsynaptic density, the neurons were transfected with a plasmid coding for the Xph20‐eGFP monobody at DIV0 to fluorescently label the postsynaptic protein PSD95 [44, 45]. Transfection was performed by electroporation using a 4D‐Nucleofector system with the P3 Primary Cell kit (Lonza), a procedure known to reduce neuronal adhesion. To compensate for electroporation induced cell loss and ensure sufficient neuronal density for long‐term maturation and synapse formation, transfected neurons were therefore seeded at a higher density, 40 000 cells/cm^2^, onto PLL‐coated Si_3_N_4_ membranes. Two hours after seeding, the membranes were transferred to a Petri dish containing an astrocyte feeder layer, then returned to the incubator. At DIV3, 2 µm AraC was added to stop astrocyte proliferation. The neurons were cultured until DIV21 to allow maturation and dendritic spine formation, with 1 mL of equilibrated NBB27 being added once per week to compensate for evaporation. In this condition, the Si_3_N_4_ membranes only contained neurons, with astrocytes remaining on the Petri dish.

### 2.2 Neuron Viability Assay

Primary rat hippocampal neurons were dissociated and cultured according to the protocol described by Kaech and Banker [43]. Viability assays were performed in neurons cultured on coverslips. Dissociated hippocampal neurons were seeded at a density of 200 000 cells in a 60 mm dish containing four PLL‐coated 18 mm coverslips. Two hours after seeding, the coverslips were transferred to a Petri dish containing a confluent astrocyte feeder layer and 5 mL of NBB27 medium. At DIV3, 2 µm AraC was added to the medium to stop astrocyte proliferation in the Petri dish, and neurons were cultured until DIV14. For metal exposure, the coverslips were transferred to a 12‐well plate containing 1 mL of culture medium from the dish with the appropriate concentration of Mn. Metal exposure was performed using the medium from the astrocyte feeder layer dish. Neurons were exposed to ultra‐pure Mn chloride (Alfa Aesar, 044442.06) for 24 h until DIV15, at different concentrations.

Neuronal viability after 24 h of Mn exposure was determined using the ReadyProbes Cell Viability Imaging Kit, Blue/Green (Invitrogen, R37609). This assay is based on the differential labeling of the nucleus according to the integrity of the plasma membrane. The NucBlue Live reagent (Hoechst 33 342) stains the nuclei of all cells (live and dead), while the NucGreen Dead reagent stains only the nuclei of dead cells. The manufacturer's protocol was adjusted to obtain optimal staining in both channels. Briefly, coverslips were placed in 12‐well plates, and the medium was replaced with 1 mL of Tyrode's solution (305 mOsm/L) just before adding 1 drop of NucBlue Live reagent and 2 drops of NucGreen Dead reagent for 45 min incubation at 37°C and 5% CO_2_. This solution was then washed and replaced with 1 mL of Tyrode's solution to remove fluorescent dyes in the solution prior to microscopy.

Microscopy was performed using a commercial video‐microscope, Leica DMI8 inverted (Leica, Germany), with a dry objective 10× (NA 0.32) and equipped with a control in temperature (37°C) and CO_2_. A CoolLED pE‐4000 illumination system (CoolLED, US) was used at 405 and 490 nm wavelengths, and an emission quad band filter DAPI/FITC/TRITC/CY5 was selected to avoid mechanical drift between the imaging of the two dyes. Images were acquired with a Flash 4.0 v2 sCMOS camera (Hamamatsu, Japan) at a 50 ms exposure time for both GFP and DAPI channels. Three replicates of each of the 8 concentrations were imaged, and for each coverslip, 7 to 10 different fields of view (1331 × 1331 µm^2^) were acquired throughout the entire coverslip.

### 2.3 Fluorescent Labeling and Mn Exposure

We only employed fluorescent probes optimized for live‐cell microscopy to circumvent the limitations of chemical fixation, which may introduce artefacts that compromise the cellular integrity and are incompatible with elemental analysis [42, 46, 47, 48, 49, 50, 51]. The fluorescence signal remained well preserved after cryo‐fixation, thereby enabling efficient localization of the specific subcellular structures of interest by cryo‐FM and prior to SXRF imaging [52].

For the ‘neuron condition’, Si_3_N_4_ membranes contained only neurons, and astrocytes were grown in the Petri dish as a feeder layer. The neurons were transduced with CellLight Golgi‐GFP (ThermoFisher Scientific, C10592) according to the manufacturer's protocol, considering a particle number of 50 per cell. At the time of transduction, the Si_3_N_4_ membranes were transferred to a 12‐well dish containing 1 mL of running medium and the required amount of CellLight Golgi‐GFP. The membranes were then incubated overnight for 16 h. The following morning, the membranes were transferred to the original Petri dish containing the astrocyte feeder layer. They were then incubated for three days without CellLight Golgi‐GFP to increase the transduction rate. After that, a fresh stock solution of Mn at 2 mm was prepared in culture medium by vortexing for three minutes and sterilized by filtration with 0.22 µm syringe filters. The Si_3_N_4_ membranes were then placed in a new Petri dish. The required amount of Mn was then added to the Petri dish to achieve a final concentration of 250 µm (IC_10_, the 10% viability inhibitory concentration). Thus, in the ‘neuron condition’, the neurons were exposed to Mn in the absence of astrocytes. Following Mn exposure and prior to cryofixation, the membranes were transferred to a 12‐well dish and incubated with 5 µg/mL of the nuclear stain Hoechst 33 342 (Sigma‐Aldrich, B1155) in equilibrated medium at 37°C for 10 min. For the ‘co‐culture condition’, the transduction and labelling procedure were similar, but in this case, Si_3_N_4_ membranes contained both cell types, astrocytes and neurons in co‐culture.

For the ‘neuron PSD condition’, neurons were initially transfected with Xph20‐eGFP and cultured until DIV21 to allow for neuronal maturation and the formation of a dense network of dendritic spines. At DIV20, a fresh stock solution of Mn at a concentration of 2 mm was prepared using equilibrated culture medium. This solution was then vortexed for three minutes and sterilized by filtration. The required amount of Mn was then added to the Petri dish to achieve a final concentration of 250 µm. Just before sample preparation by cryofixation at DIV21, the neurons were labelled with SiR‐tubulin (silicon rhodamine tubulin, Spirochrome, SC002) to fluorescently label microtubules [41]. To minimize the amount of reagent, the Si_3_N_4_ membranes were transferred into a 12‐well dish containing 0.5 mL of equilibrated culture medium and 1 µm of SiR‐tubulin. The neurons were then cultured for 1 h at 37°C.

### 2.4 Sample Cryo‐Processing for SXRF Analysis

To preserve the cell structure and chemical distribution, the samples were cryopreserved in accordance with the methods previously published [39, 40]. Briefly, after labelling and Mn exposure, the samples were plunged into liquid ethane cooled to ‐140°C with liquid nitrogen. This process was carried out using an automated vitrification system (Vitrobot Mark IV) from FEI (Thermo Fisher, USA). Immediately prior to vitrification, the membranes were gently and rapidly immersed in a warmed ammonium acetate buffer solution (adjusted to a pH of 7.4 and a concentration of 240 mOsm, the same as the culture medium and prepared using ultra‐trace grade water) to remove the extracellular inorganic salts present in the culture medium. Shortly after washing, the samples were placed in the Vitrobot system, where the excess ammonium acetate buffer solution was automatically blotted (blotting parameters: 3 s, force 2, 2 blots) using ashless, ultra‐absorbent filter paper (Ted Pella, 47000, 595). The membranes were then freeze‐plunged into chilled liquid ethane to form a thin layer of ice compatible with cryo‐FLM (Fluorescence Light Microscopy). After cryofixation, the samples were stored in cryogenic tubes in liquid nitrogen.

After imaging by cryo‐FLM (see the next section), the samples were freeze‐dried using a Christ Alpha 2‐4 LD Plus freeze dryer at −75°C for 48 h under primary vacuum (2 × 10^−^^3^ mbar). The freeze‐dried samples were then allowed to gradually reach room temperature under vacuum conditions for 5 h. Once room temperature was reached, the vacuum was released, and dry air from desiccant silica beads was allowed to enter. Finally, the freeze‐dried samples were stored in a desiccant cabinet at room temperature until SXRF analysis.

### 2.5 Cryo‐Fluorescence Light Microscopy (Cryo‐FLM)

The cryo‐fixed membranes were transferred to a Leica (Germany) commercial wide‐field EM‐Cryo‐CLEM microscope at the Bordeaux Imaging Centre (BIC). This system keeps the samples in liquid nitrogen vapor throughout the entire process, including transfer, storage, and observation. Cryo‐FLM images were acquired using Metamorph software (Molecular Devices). The physical length of the images was set to 2304 pixels at 104 nm/pixel using an HC PL APO 50×/0.90 NA cryo objective and an Orca‐Fusion BT camera (Hamamatsu Photonics France SARL).

Golgi and Hoechst 33 342 imaging was performed using a GFP filter (470/40 nm excitation, BP 525/550) and a DAPI filter (360/40 nm excitation, BP 470/40 nm), respectively. The same GFP filter and a Y5 filter (620/60 nm excitation, BP 700/75 nm) were used for GFP‐xph20 and SiR‐tubulin imaging. For each wavelength, a z‐stack with a thickness of 3–4 µm was acquired in order to image the full cell volume. Bright‐field images were also acquired for the same regions to improve final correlation.

After freeze‐drying the samples, the same neurons or astrocytes were imaged again at room temperature using the DAPI filter and brightfield illumination to map the entire membrane and facilitate identification of the regions of interest during SXRF imaging. Thanks to their different morphology, neurons and astrocytes can be distinguished by optical microscopy. See Supporting Information S1 for details of the correlative protocol.

### 2.6 Synchrotron X‐Ray Fluorescence Imaging

SXRF imaging experiments were performed at the ID21 beamline of the ESRF (European Synchrotron Radiation Facility) using two experimental setups. In the first setup, a monochromatic beam of 7.4 keV was focused to 890 nm × 230 nm, providing a flux of 1.1 × 10^1^ ^1^ photons/s. Samples were raster scanned in continuous mode with an integration time of 100 ms/pixel and a step size of 500 nm/pixel. The larger spatial resolution enabled a significant number of whole cells to be analyzed for each condition and cell type, providing statistically significant information [53]. In the second setup, the recently commissioned nanoscope at ID21 was used for imaging dendritic spines at room temperature and under vacuum conditions. This new setup enabled a 10.2 keV monochromatic beam to be focused down to a size of 167 nm × 151 nm, providing a flux of 9.9 × 10^10^ photons/s. Samples were raster scanned continuously with an integration time of 100 ms/pixel and a step size of 200 nm/pixel.

The XRF signal was recorded using X‐ray detectors based on multi‐element silicon drift diodes, which were positioned at 90° to the incident beam. The summed X‐ray fluorescence spectra from these detectors were fitted using Python scripts and the PyMCA library [54], and were calibrated against the signal derived from a thin‐film X‐ray fluorescence standard (AXO Dresden GmbH, Germany). The resulting areal mass density distributions of the detected chemical elements were saved individually as 32‐bit TIFF images.

For element quantification, two regions of interest (ROIs) were defined in the quantitative images using FIJI software [55]: one ROI from the neuron and one for the background. The intracellular element content, expressed in ng/mm^2^, was calculated as the difference between the mean intensity of the two ROIs (see Supporting Information S2). These values were then converted to micromolar units (see Supporting Information S3a) by measuring the thickness of living neurons and astrocytes using a calibrated Z microscope (see Supporting Information S4). The elemental content was then compared across two conditions: neurons exposed to Mn in the absence of astrocytes (the ‘neuron‐alone’ condition); neurons and astrocytes exposed to Mn in co‐culture (the ‘co‐culture’ condition). Quantitative analyses were performed during synchrotron session LS3142, to compare the ‘neuron‐alone’ and ‘co‐culture’ conditions, while the synaptic imaging was achieved during session LS3418, to image neurons transfected with Xph20‐eGFP. For quantitative analyses: for the ‘neuron‐alone’ condition, 23 neurons were analyzed on two different SN membranes from two independent cell cultures; for the ‘co‐culture’ condition, 15 neurons and 19 astrocytes were analyzed on two different SN membranes from two independent cell cultures. Samples for each condition were prepared in triplicate but only biological duplicates could be analyzed due to analytical time constraints during synchrotron sessions.

### 2.7 Correlative Imaging

For correlative imaging purposes, the Icy software [56] version 2.4.3 (http://icy.bioimageanalysis.org/) and the eC‐CLEM plugin [57] were used. Cryo‐FLM images were presented as the maximum projection of the acquired z‐stack to enable superposition with XRF maps. The procedure for correlating cryo‐FLM and SXRF images is outlined in Supporting Information S1. Three groups of images were superimposed. The first group comprised the cryo‐FLM images (Golgi, nucleus, and bright field); the second comprised the images of freeze‐dried samples (bright field, nucleus, and Golgi); and the third comprised the SXRF images of the chemical elements.

### 2.8 Statistical Analysis

The statistical analysis of the cell viability assays was performed in two steps. First, the microscopy images were analyzed using an ImageJ macro to segment the cells in the two measurement channels: total cells and dead cells. These results were then processed using Rstudio Notebooks to perform statistical analysis, plot dose‐response curves and to calculate IC_10_ and IC_50_ values (10% and 50% viability inhibitory concentrations). Three dose‐response models were employed, and the weighted mean was calculated to plot the final dose‐response curve and determine the IC values. R software (version 4.3.0) and [58]. (version 12.0), Tidyverse (2.0.0) [59] and drc (3.0‐1) [60] R packages were used.

GraphPad Prism 8.4.3 for Windows (Boston, Massachusetts, USA; www.graphpad.com) was used for the statistical analysis of SXRF quantitative data to produce scatter plots (lines in the scatter plots represent the median and the confidence interval at 95%), and the associated statistics (Supporting Information S5). The number of analyses in each group was *n* = 23, 15, and 19 for ‘neurons alone’, ‘co‐cultured neurons’, and ‘co‐cultured astrocytes’ conditions, respectively. Data were normalized by the mean volume of the cells, neurons, or astrocytes, as explained in Section 3.3 and Supporting Information S3a. Outliers were identified and removed using the ROUT methods with a false discovery rate (q) set at 0.1% (Supporting Information S3b). Normality of the data was assessed using the Shapiro–Wilk test. Since the data did not follow a normal distribution (*p* < 0.05), the non‐parametric Mann–Whitney *U*‐test was used for further statistical analysis (Supporting Information S5).

## 3 Results

### 3.1 Manganese Subcellular Imaging in Neurons and Astrocytes

**FIGURE 1:**
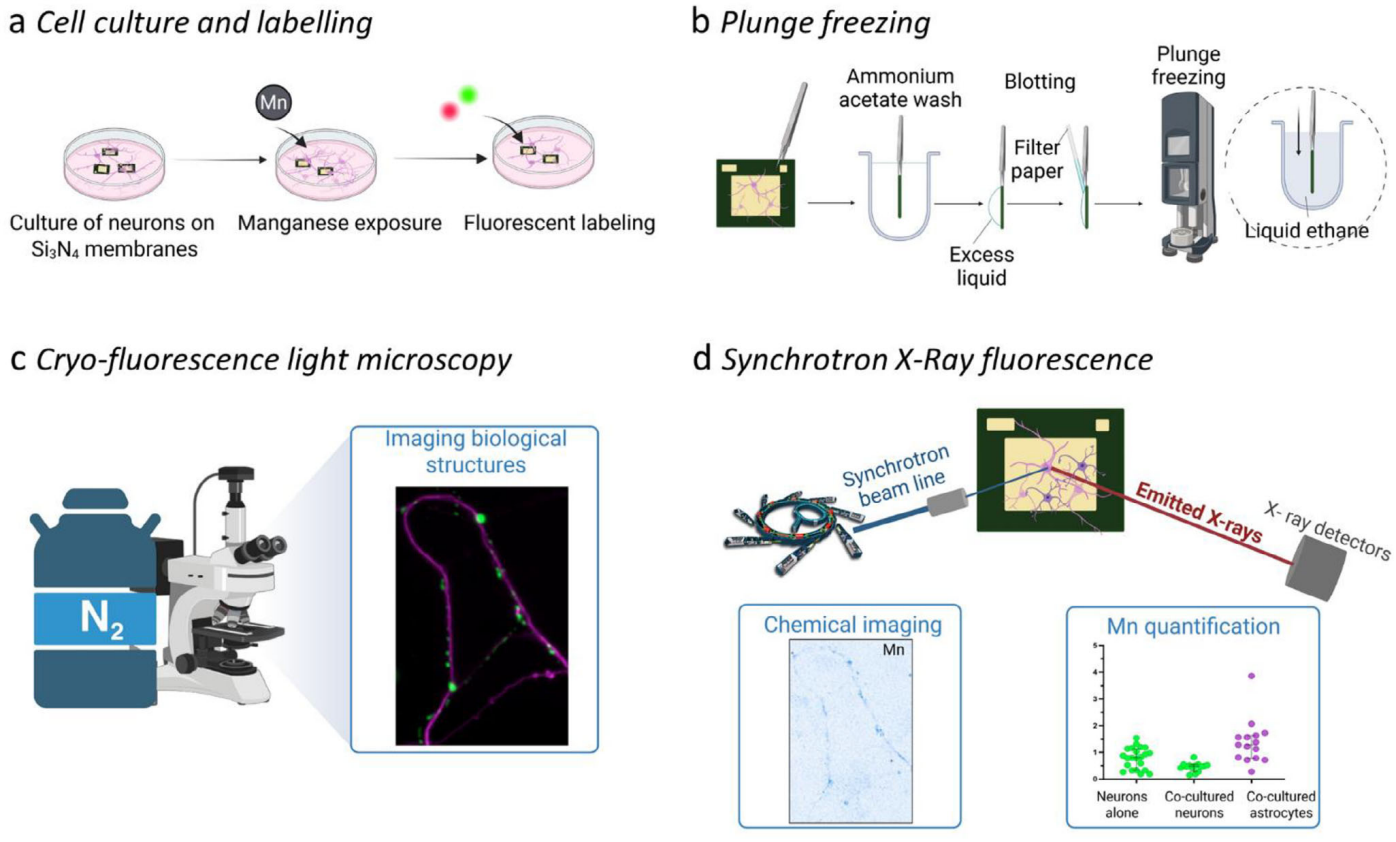
Schematic overview of the sample preparation and correlative imaging workflow combining cryo‐FLM and synchrotron XRF imaging. (a) Primary cultures of hippocampal neurons and astrocytes onto silicon nitride membranes, exposed to Mn, and fluorescently labeled. (b) After labeling, the samples were rapidly plunge‐frozen in liquid ethane using an automated device. (c) Cryo‐FLM was performed to visualize the distribution of fluorescently labeled cellular structures on cryofixed samples. (d) The same regions of interest were analyzed by SXRF for both correlative imaging purposes and element quantification. Created with BioRender.com.

As described in Section 2.3, we employed fluorescent probes optimized for live‐cell labeling to circumvent the limitations of chemical fixation. The locations of the fluorescent markers of interest were imaged by cryo‐FLM and their positions recorded. Subsequently, the same cells were analyzed using SXRF to visualize the distribution of chemical elements (Figure 1).

**FIGURE 2:**
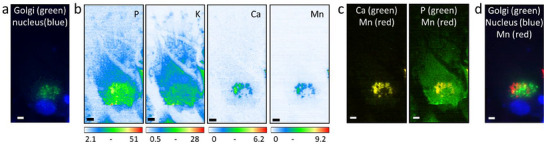
Representative example of organelle and elemental imaging in astrocytes after Mn exposure. (a) Merged images of the nucleus (blue) and the Golgi apparatus (green) obtained by cryo‐FLM. (b) Elemental distribution of P, K, Ca, and Mn obtained by SXRF, scan size 48 µm × 95 µm, step 0.5 µm/pixel, scan time 100 ms/pixel. The color scales represent the quantitative values per pixel, expressed in ng mm^−2^. (c) Merged images of Ca (green) and Mn (red), showing a colocalization of both elements (yellow), and of P (green) and Mn (red) showing Mn is restricted to the cytoplasm. (d) Merged images of Mn (red), Golgi apparatus (green), and nucleus (blue). Scale bars: 5 µm.

In control cells, not exposed to Mn, the quantification of this element was below the detection limit and could not be imaged in astrocytes or neurons (see Supporting Information S6). An example of the elemental distribution in a cultured astrocyte from the ‘co‐culture condition’ exposed to 250 µm of Mn for 24 h (corresponding to IC_10_, see Supporting Information S7) is shown in Figure 2; other examples can be found in Supporting Information S8. The cryo‐FLM image shows the Golgi apparatus located in a compact cytoplasmic area close to the nucleus (Figure 2a). The elemental distribution obtained by SXRF imaging shows that phosphorous (P) and potassium (K) are distributed throughout the cell, whereas calcium (Ca) and Mn are located in a compact area of the cytoplasm (Figure 2b). Merging the elemental distributions reveals that Mn co‐localizes with Ca and P in the cytoplasm (Figure 2c). Merging cryo‐FLM and SXRF images allows obtaining the distribution of the chemical elements and organelles to be visualized together. The Mn distribution (red) partially co‐localizes with the green fluorescence of the Golgi apparatus, adjacent to the nucleus (blue) (Figure 2d). This only partial co‐localization of Mn and Golgi apparatus could be explained by differences in protein localization at the sub‐compartment level of the Golgi apparatus (see Discussion and Conclusion).

**FIGURE 3:**
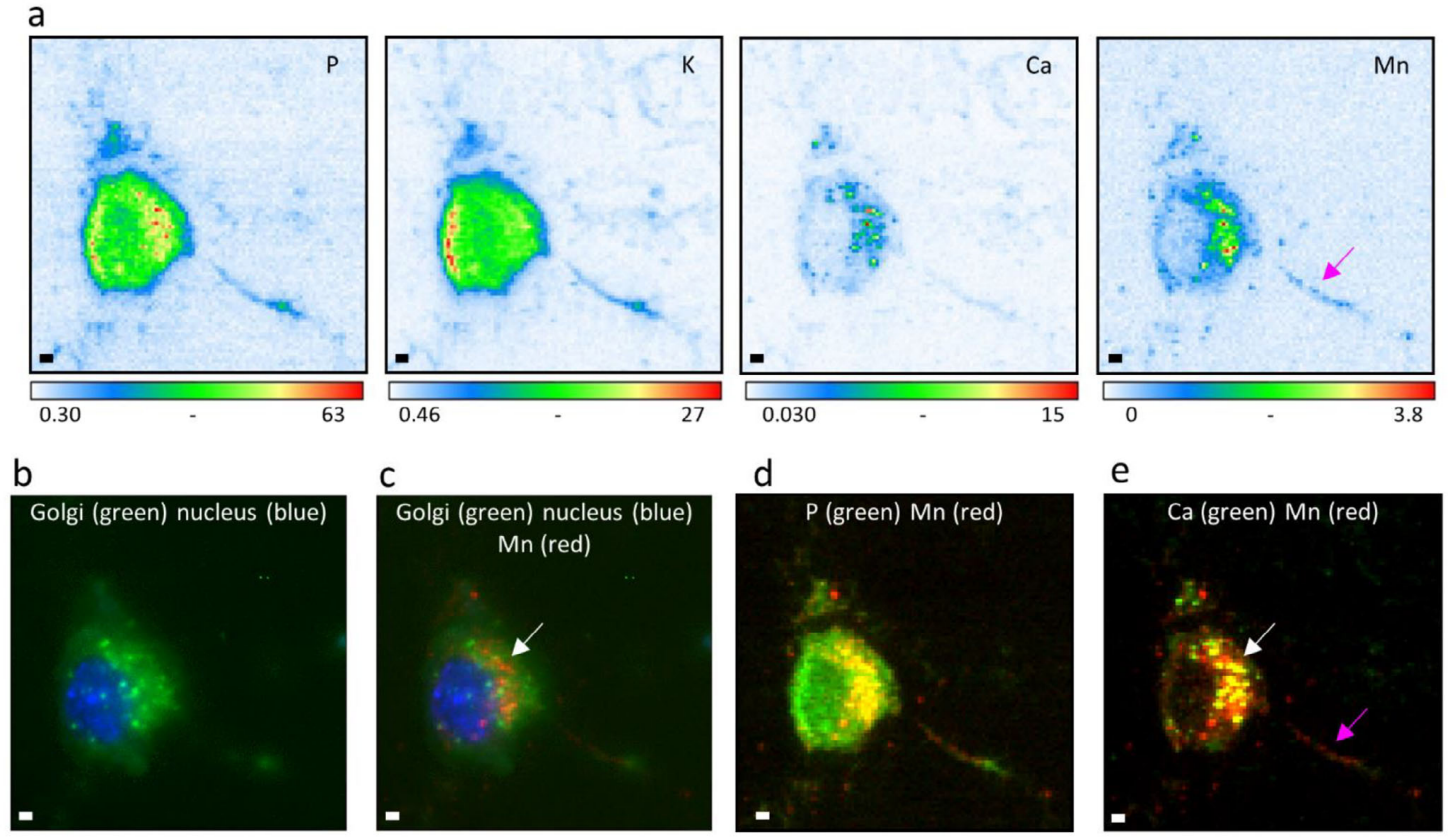
Representative example of organelle and elemental imaging in primary neurons after Mn exposure in the presence of astrocytes. (a) Elemental distribution of P, K, Ca, and Mn obtained by SXRF, scan size 50 µm × 50 µm, step 0.5 µm/pixel, scan time 100 ms/pixel. The magenta arrow points a dendritic extension. The color scales represent the quantitative values per pixel, expressed in ng mm^−2^. (b) Merged images of the nucleus fluorescence (blue) and the Golgi apparatus (green) obtained by cryo‐FLM. (c) Merged images of Mn (red), Golgi apparatus (green) and nucleus (blue), showing the presence of Mn in the Golgi region (white arrow). (d) Merged images of P and Mn, showing that most of the Mn is within a restricted area of the cytoplasm. (e) Merged images of Ca and Mn, showing colocalization of both elements in the Golgi region (yellow, white arrow) and Mn reaching the dendritic extension (magenta arrow). Scale bars: 5 µm.

Figure 3 shows an example of the elemental distribution in a primary hippocampal neuron exposed to Mn in the presence of astrocytes (‘co‐culture condition’). Other examples can be found in Supporting Information S9. The elemental distribution shows that P and K are distributed throughout the cell, while Ca and Mn follow a dot‐like distribution and are principally located on the right side of the cell (Figure 3a). The cryo‐FLM images reveal a dot‐like distribution of the Golgi apparatus (green) on the right side of the nucleus (blue) (Figure 3b). Superimposing the cryo‐FLM and SXRF images with Mn (red) reveals that Mn is located in the Golgi apparatus region (white arrow) (Figure 3c), although the individual vesicles do not show strict colocalization. Superposition of Mn (red) and P (green) shows that Mn is restricted to a cytoplasmic sub‐region on the right side of the neuron (Figure 3d). Mn (red) is colocalized with Ca (green) where the Golgi apparatus is located (white arrow) (Figure 3e), similarly to the results found for astrocytes (Figure 2c). Interestingly, outside the cytoplasmic region, some Mn vesicles are found along the neuronal extensions (magenta arrows in Figure 3a,e).

**FIGURE 4:**
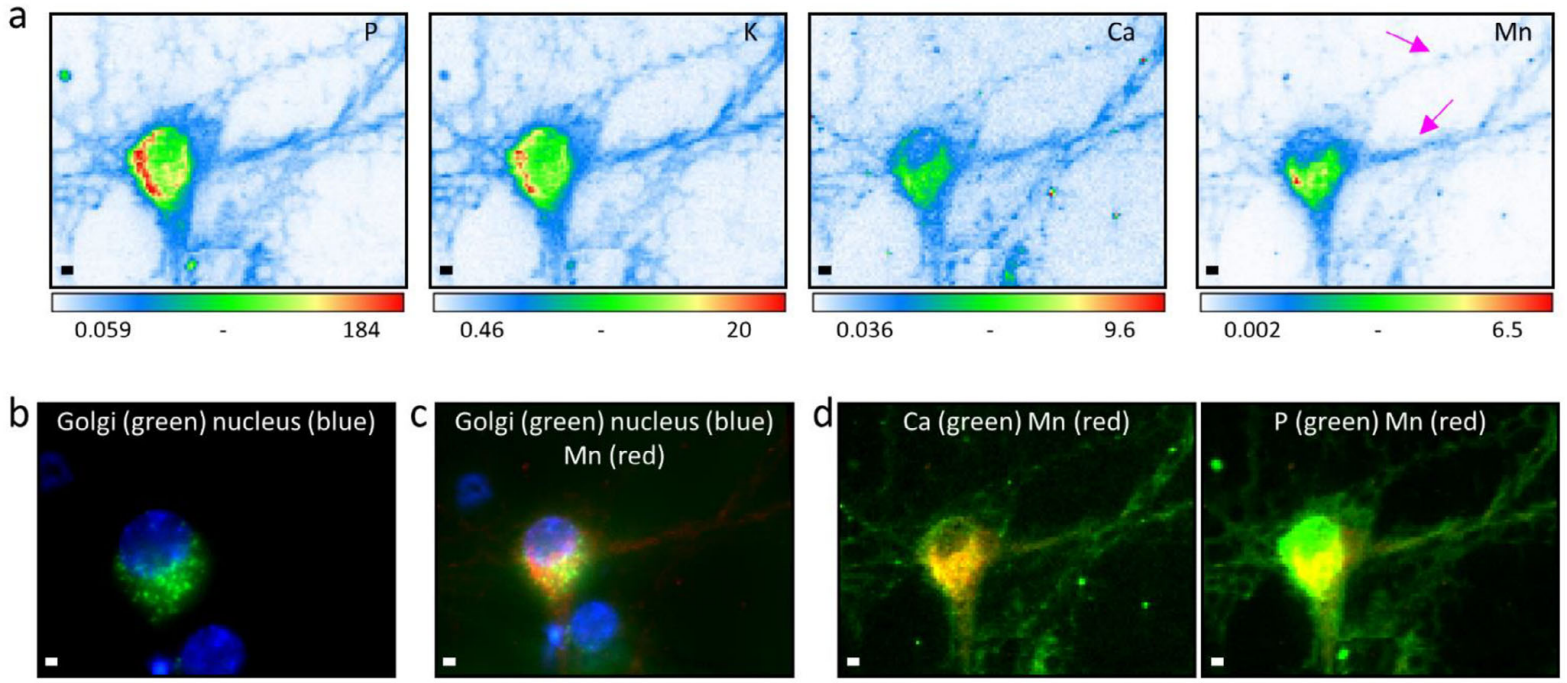
Representative example of organelle and elemental imaging in primary neurons after Mn exposure in the absence of astrocytes. (a) Elemental distribution of P, K, Ca, and Mn obtained by SXRF, scan size 60 µm × 46 µm, step 0.5 µm/pixel, scan time 100 ms/pixel. The magenta arrows point dendritic extensions. The color scales represent the quantitative values per pixel, expressed in ng mm^−2^. (b) Merged images of the nucleus (blue) and the Golgi apparatus (green) obtained by cryo‐fluorescence light microscopy. (c) Superimposed images of Mn (red), Golgi (green), and nucleus (blue). (d) Merged images of Ca and Mn, showing colocalization of both elements (yellow), and merged images of P and Mn, showing Mn in a restricted area of the cytoplasm. Scale bars: 2 µm.

Figure 4 shows an example of the elemental distribution in a primary hippocampal neuron exposed to Mn in the absence of astrocytes (‘neuron condition’). Other examples can be found in Supporting Information S10. Figure 4a shows the elemental distributions, with P and K distributed throughout the cell and Ca and Mn concentrated in dot‐like structures. The cryo‐FLM images (Figure 4b) show the Golgi apparatus displaying a punctate pattern beneath the nucleus. Superimposing the cryo‐FLM and SXRF images (Figure 4c) reveals that Mn is located in the Golgi region (Figure 4c; Supporting Information S10). Superposition of Ca and Mn (Figure 4d) shows that both elements colocalize in the Golgi region. Superposition of P and Mn confirms that Mn is restricted to a cytoplasmic region (Figure 4d). Interestingly, outside the cytoplasmic region, some Mn is found along the dendrites, as observed in neurons from both the ‘co‐culture’ and ‘neuron’ conditions (Figures 3a and 4a, magenta arrows).

**FIGURE 5:**
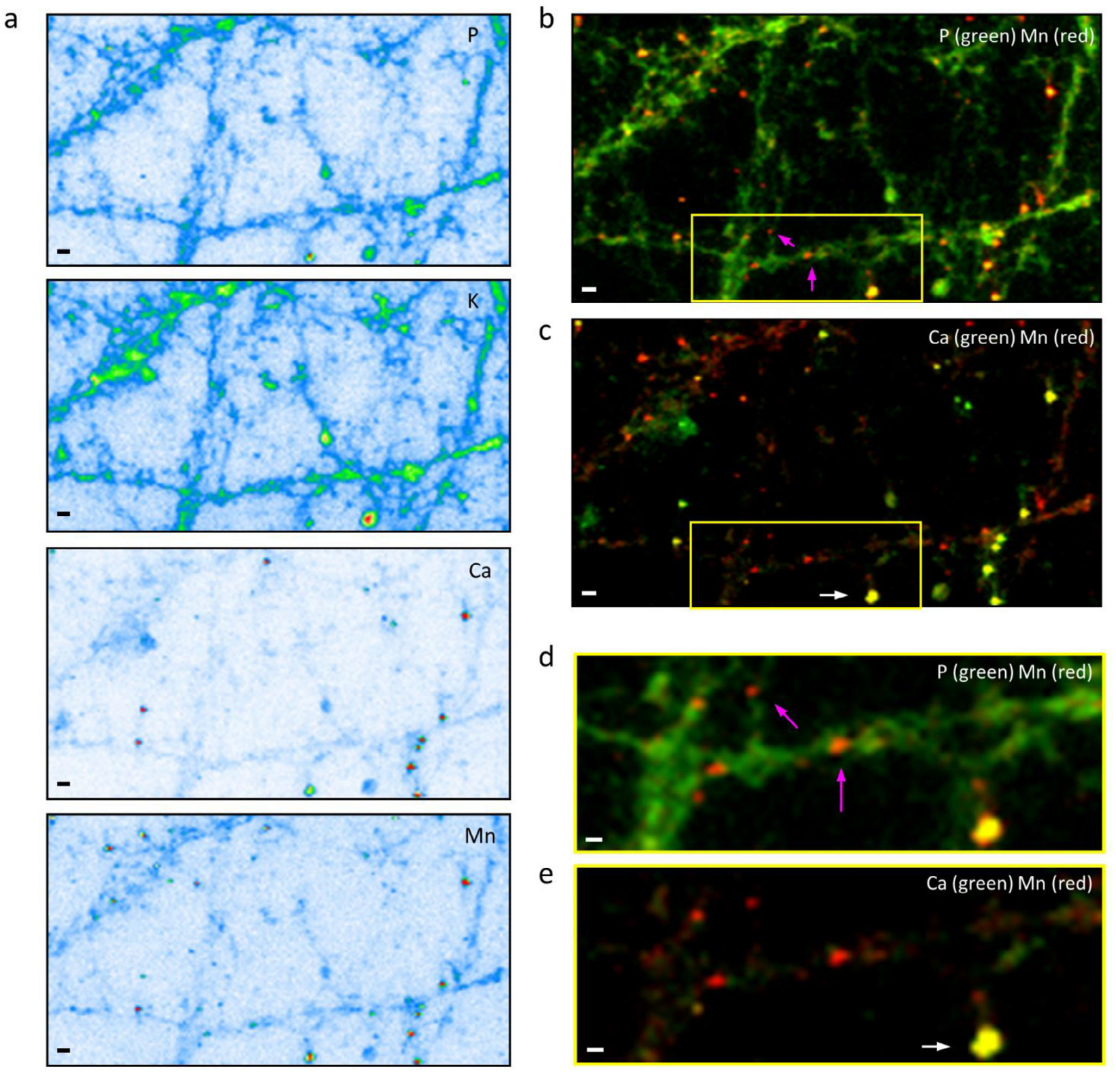
Representative example of elemental imaging in the dendritic network of hippocampal neurons. (a) Elemental distribution of P, K, Ca, and Mn obtained by SXRF, scan size 74 µm × 40 µm, step 0.4 µm/pixel, scan time 100 ms/pixel. (b) Merged images of Mn and P, showing Mn dot‐like distribution along dendrites (magenta arrows). (c) Merged images of Mn and Ca showing some co‐localization within large puncta (white arrow). (d) Zoomed region from the yellow square in (b) showing Mn along dendrites. (e) Zoomed region from the yellow square in (c) showing Mn and Ca colocalization. Scale bar 2 µm (a–c); 1 µm (d,e).

To investigate whether Mn reaches regions other than the Golgi apparatus in neurons, we imaged dendrites far from the soma (Figure 5). The morphology of neuronal extensions is displayed by the P and K distributions, two major intracellular elements, while Ca and Mn show a dot‐like distribution (Figure 5a) (see also Supporting Information S11). Merged images of P (green) and Mn (red) show that Mn dots are scattered across neuronal extensions (magenta arrows) (Figure 5b and zoomed area in Figure 5d). The merged images of Ca and Mn reveal that large Mn puncta colocalize with Ca (white arrows), while smaller ones do not (Figure 5c and zoomed area in Figure 5e). SXRF imaging shows that Mn localization is not restricted to the Golgi region, it reaches neuronal extensions forming dot‐like structures, and that the large Mn‐rich structures colocalize with Ca.

### 3.2 High Resolution Manganese Imaging in Dendritic Spines

**FIGURE 6:**
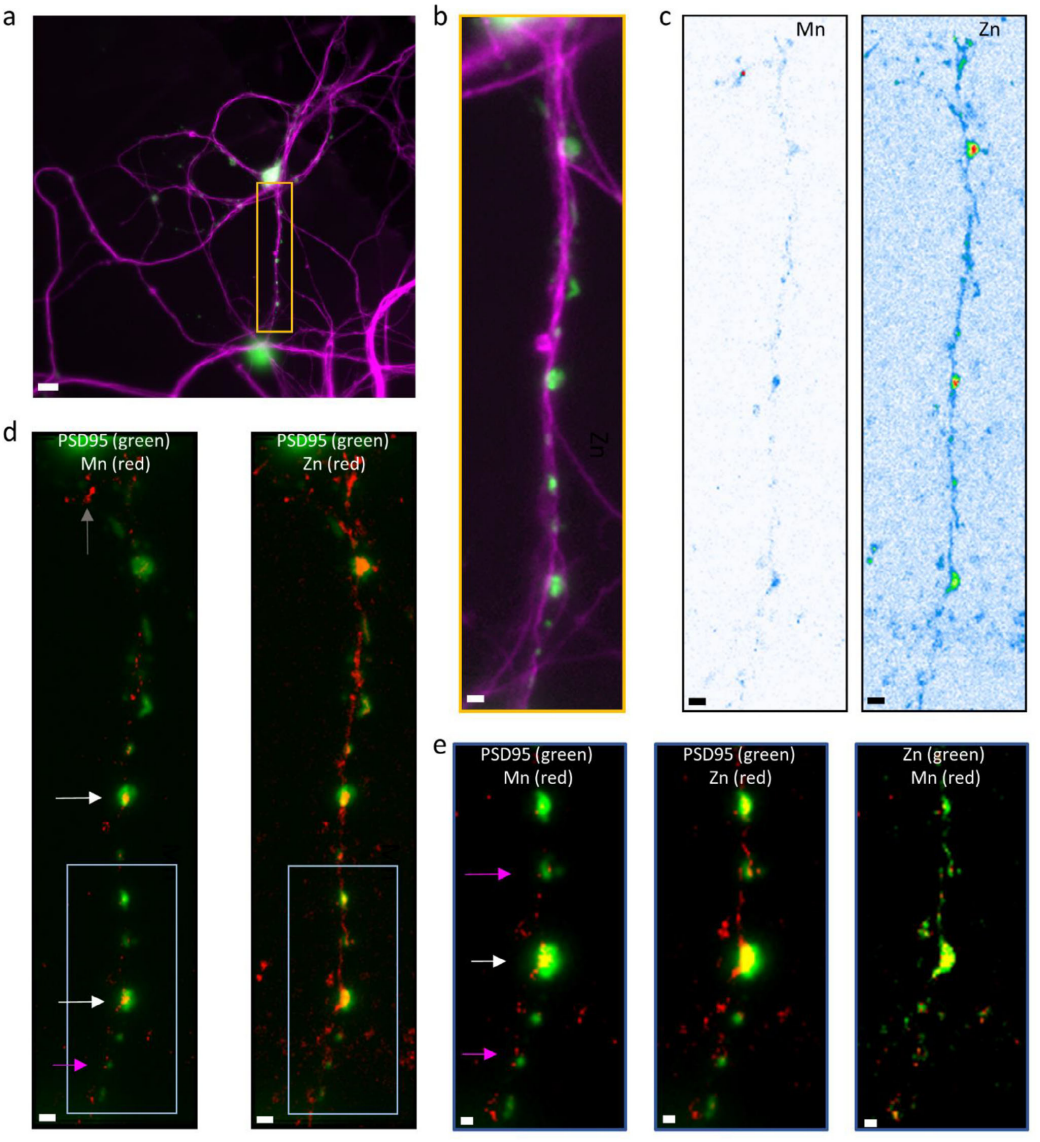
Representative example of elemental imaging in dendritic spines of primary neurons exposed to Mn. (a) Confocal fluorescence images of tubulin (magenta) and PSD‐95 (green) in living primary hippocampal neurons at DIV21. (b) Cryo‐FLM images of the framed region in a, showing fluorescence of tubulin (magenta) and PSD‐95 (green) in immobilized cryogenic conditions. (c) Elemental distribution of Zn and Mn obtained by SXRF, scan size 85 µm × 20 µm, step 0.2 µm/pixel, scanning time 100 ms/pixel. (d) Superimposed images of Mn (red) and PSD‐95 (green) distribution and of Zn (red) and PSD‐95 (green) showing Mn reaching the postsynaptic density (white arrows), in the close vicinity of the postsynaptic density (magenta arrows), and along the microtubules (gray arrow). (e) Zoom in the region framed in d to show Mn and Zn location, respectively to PSD‐95, and Mn and Zn superposition. Scale bars: 10 µm (a); 2 µm (b–d); 1 µm (e).

To investigate whether Mn reaches dendritic spines, we fluorescently labelled the PSD‐95 protein to localize post‐synaptic compartments and the tubulin protein to image microtubules (Figure 6; Supporting Information S12). Figure 6a shows an overview of the neuronal network. PSD‐95 fluorescence was observed along the microtubules in the selected region of interest. These areas were imaged using cryo‐FLM to discern the distribution of tubulin (magenta) and PSD‐95 (green) (see Figure 6b). The same region was analyzed using SXRF imaging to determine the distribution of elements such as Mn and Zn (Figure 6c). Using ICY and eC‐CLEM (Section 2.7), the cryo‐FLM and SXRF images were superimposed to reveal the overlap between the distribution of PSD‐95 and the localization of Mn and Zn (Figure 6d).

**FIGURE 7:**
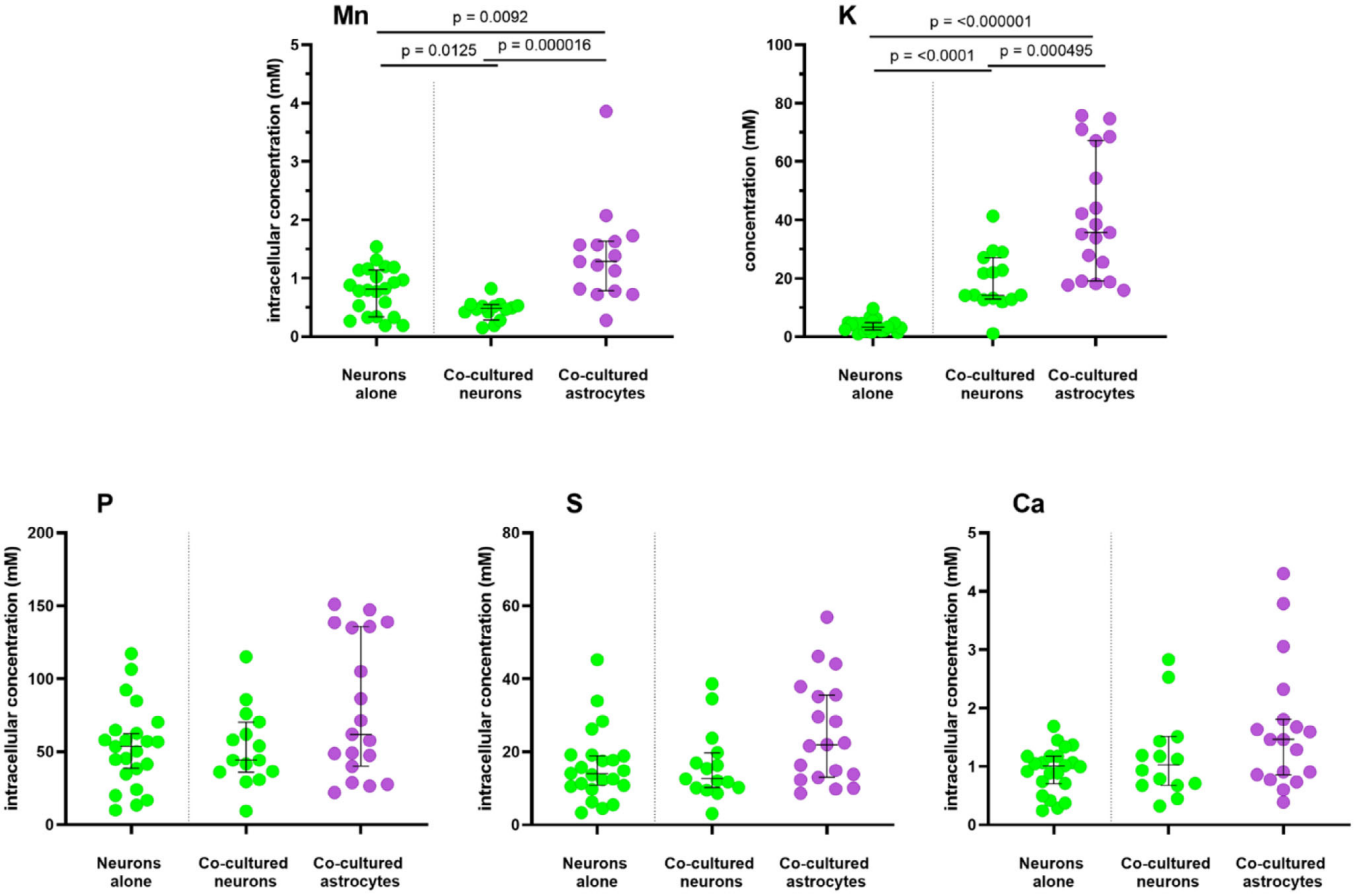
Elemental content in neurons and astrocytes after Mn exposure. Each dot in the scatter plot represents the quantitative values obtained for one neuron (green) or astrocyte (magenta). For each condition, the black lines represent the median and the confidence interval at 95%. Data did not fulfil the Shapiro–Wilk test and groups and were compared using the Mann–Whitney statistical test. The number of cells measured for each group was as follows: 23, 15, and 19 for neurons alone, co‐cultured neurons, and co‐cultured astrocytes, respectively. Outliers were removed. *p*‐values are indicated in the figure. For details on statistical data analysis, see Section 2.8 and Supporting Information S5.

Three types of Mn distribution can be distinguished from the superimposed images (Figure 6d; Supporting Information S12d). First, Mn is present in the highly fluorescent PSD‐95 spots (Figure 6d, white arrow, and Supporting Information S12e). Second, Mn is found in close proximity to small PSD‐95 spots (Figure 6d, magenta arrows, and Supporting Information S12f). Third, some larger Mn spots are present along microtubules (Figure 6d, grey arrow, and Supporting Information S12g). This is clearly visible in the zoomed regions (Figure 6e; Supporting Information S12e–g): Mn reaches large postsynaptic compartments (Figure 6e white arrow), or is located around smaller postsynaptic compartments (Figure 6e magenta arrows). Zn is also present in the postsynaptic compartments (Figure 7e; Supporting Information S12e–g), as expected from our previous findings regarding Zn distribution in neurons [38]. Finally, the superposition of Zn and Mn indicates that the two elements are not strictly colocalized, although both elements are located within the larger PSD‐95 fluorescent structures, or around the smaller ones (Figure 7e; Supporting Information S12e–g).

### 3.3 Multi‐Element Quantification in Neurons and Astrocytes

Two types of cell exposure to Mn were compared. In the ‘neuron condition’, the neurons were cultured alone on the Si_3_N_4_ membranes and in close contact with a feeder layer of astrocytes grown on the Petri dish, except during Mn exposure, which was conducted without the astrocyte layer (see Section 2.1). In the ‘co‐culture condition’, neurons and astrocytes were co‐cultured on the Si_3_N_4_ membranes during the entire process, including Mn exposure. Mn exposure was set to 250 µm Mn for 24 h, corresponding to the IC_10_ viability assay. Element quantification was compared on three groups: (1) neurons cultured alone from ‘neuron condition’, (2) neurons cultured with astrocytes from ‘co‐culture condition’, and (3) astrocytes from ‘co‐culture condition’.

The intracellular content, as calculated by SXRF data analysis, is initially expressed in ng mm^−2^. As explained in Section 2.6 and Supporting Information S3a, these values were transformed into mm units. To do this, the thickness of each cell type, 15.5 ± 2.3 µm for neurons and 5.8 ± 0.8 µm for astrocytes, was measured in living cells (Supporting Information S4). Therefore, the expressed concentrations in mm are volumetric and take into account the volume under hydrated conditions.

With regard to Mn quantification, a significant difference was observed between neurons exposed to Mn in the presence or absence of astrocytes (*p* = 0.0125). The median and median absolute deviation for neurons were 480 ± 60 and 810 ± 340 µm, respectively (see Figure 7; Supporting Information S3b). Mn accumulation is almost halved in neurons from the ‘co‐culture condition’ compared to neurons from the ‘neuron condition’, with a 40% decrease of the median value and 43% decrease of the mean (see Figure 7; Supporting Information S3b). Astrocytes have a higher capacity to accumulate Mn than neurons, with a median and median absolute deviation of 1290 ± 440 µm, representing 270% of the median Mn content in co‐cultured neurons and 300% of the mean value (see Figure 7; Supporting Information S3b).

Our results also show statistically significant changes in potassium content. There is a lower potassium content in both neuronal populations compared to astrocytes (Figure 7). Moreover, in the ‘neuron condition’, potassium content in neurons is statistically significantly lower than in neurons from the ‘co‐culture condition’. This result may indicate that the integrity of neurons is compromised resulting in potassium loss when they accumulate more Mn. The intracellular accumulation of other elements such as P, S, and Ca is not statistically different between neurons and astrocytes and is neither modified by the presence or absence of an astrocyte layer (Figure 7).

## 4 Discussion and Conclusion

Mn is an essential element found at trace levels in human tissues, with an estimated total amount of 390 µg in the adult human brain [61]. This typically corresponds to 0.3 µg/g of wet weight ([62] or 1.3 µg/g of dry weight [63]. Due to this low concentration, detecting and imaging Mn in single cells is challenging and requires highly sensitive spatially resolved analytical techniques. Due to such analytical challenges, imaging endogenous levels of Mn (<1 µg/g) in subcellular compartments is below the detection limit of current micro‐analytical techniques, as was found in this study when using SXRF (Supporting Information S6). However, Mn subcellular imaging can be achieved on cells supplemented with Mn. Probably due to its essential functions, Mn is toxic only at relatively high extracellular concentrations. In this study, we determined that the IC_10_ for primary rat hippocampal neurons was 250 µm (Supporting Information S7). This value falls within the same concentration range as that obtained for cell lines in previous studies [64, 65, 66].

After Mn exposure, only a few studies have addressed the subcellular distribution of Mn in brain cells or, more generally, in mammalian cells to date. This has been achieved by experimental approaches with high spatial resolution and high analytical sensitivity, such as synchrotron XRF [65, 67]; or with the recent development of chemical biology tools to image Mn^2+^ ions in living cells [68, 69]. These fluorescent biomolecular sensors are of great promise for understanding dynamic processes involving Mn^2+^ ions in cells, but they are not yet commercially available and have not yet been used in brain cells. Moreover, they are restricted to imaging labile Mn^2+^ ions and cannot image the total cellular pool of Mn, which includes Mn bound to biomolecules.

Our results provide new data showing that the Golgi apparatus is the main storage organelle for Mn in neurons and astrocytes (Figures 2, 3, 4; Supporting Information S8–S10), confirming a mechanism that has been observed for other cell types [65, 70, 71, 72]. Using correlative synchrotron XRF imaging and fluorescence microscopy, we previously demonstrated that the main subcellular site of Mn accumulation in neuron‐like PC12 rat cells exposed to Mn was the Golgi apparatus [65, 71]. Mn^2+^ ions and total Mn were located within the Golgi apparatus in HEK293T primary human embryonic kidney cells [72]. We have also observed Mn accumulation in the Golgi apparatus of HeLa human cervical carcinoma cells overexpressing a mutant SLC30A10 protein [70]. Synchrotron XRF imaging of primary mouse hippocampal neurons and of neuron‐like N2a mouse cells exposed to Mn showed that Mn was mainly located in perinuclear areas resembling the Golgi apparatus [73], in terms of size, shape, and subcellular localization. However, strict identification of the organelles was not performed. A similar result was obtained using synchrotron XRF imaging on primary mouse midbrain neurons exposed to Mn, which showed perinuclear Mn accumulation [74, 75]. In our study, we imaged the distribution of Mn and the localization of the Golgi apparatus. We report their co‐localization in primary rat hippocampal neurons and astrocytes exposed to Mn and demonstrate that the Golgi apparatus is the main organelle of Mn accumulation in these brain cells.

The accumulation of Mn in the Golgi apparatus coincides with the localization of the secretory‐pathway Ca^2+^‐ATPase SCPA1, which is known to transport Ca and Mn ions from the cytosol to the Golgi apparatus [76]. SCPA1 can transport both Ca^2+^ and Mn^2+^ ions and is expressed mainly in the trans‐Golgi network [77]. Our results demonstrate that Mn co‐localizes with Ca in the Golgi apparatus region (Figures 2, 3, 4; Supporting Information S8–S10). Furthermore, we found that the distribution of Mn/Ca does not correlate strictly with the localization of the CellLight Golgi‐GFP marker (Figures 2, 3, 4; Supporting Information S8–S10). This can be explained by the cis‐Golgi localization of the N‐acetylglucosaminyltransferase used in the CellLight Golgi‐GFP construct [78], which differs from the trans‐Golgi localization of SCPA1. Our results strongly suggest that SPCA1 and the Golgi apparatus play an important role in Mn storage and neuroprotection. This is consistent with the observation that HeLa cell viability increases when SPCA1 activity is increased [79]. In glial cells, SPCA1 protects against Mn toxicity, increasing cell viability in overexpressing cells and decreasing it in silenced cells [80]. At the organ level, magnetic resonance imaging of animals exposed to Mn has shown Mn accumulation in brain regions with high SPCA1 expression, such as the CA3 region of the hippocampus [81]. However, following exposure to high levels of Mn, this protective mechanism may become overwhelmed, resulting in Mn toxicity and impairment of Golgi apparatus functions [65, 80]. For example, high levels of exposure to Mn lead to fragmentation of the Golgi apparatus and loss of the Golgi ribbon structure in neurons and glial cells [80, 81]. Golgi fragmentation is a common feature of many neurodegenerative diseases, including Alzheimer's disease, Huntington's disease, amyotrophic lateral sclerosis, and Parkinson's disease [82]. Therefore, the Golgi apparatus is involved in both neuroprotection, through Mn storage, and neurotoxicity, when its structure begins to deteriorate, resulting in altered Golgi functions and neurodegeneration.

Although most of the Mn reaches the Golgi, other organelles and subcellular regions may also accumulate Mn in smaller amounts, which may help explain the pleiotropic neurotoxic effects of Mn. Our results demonstrate that Mn can reach the postsynaptic compartment, potentially triggering toxic effects (see Figure 6; Supporting Information S12). There were some indications in the literature that Mn could accumulate in neurites. Synchrotron XRF imaging has observed discrete Mn spots along neurites in primary mouse hippocampal neurons [73] and primary mouse midbrain neurons [75], although the exact localization of this Mn was not characterized further. Our finding is consistent with a recent study demonstrating that exposure to Mn in mice leads to significant Mn accumulation in the hippocampus, resulting in neuronal damage and reduced dendritic spine density [18]. This is also consistent with electron microscopy observations in mice treated with Mn, which showed decreased electron density in postsynaptic areas [83]. Compared to zinc distribution, which is physiologically enriched in the PSD [38, 84], Mn displays a different distribution within the PSD (see Figure 6; Supporting Information S12). While zinc fills the fluorescently labelled PSD region, Mn accumulates in smaller subregions. This result indirectly suggests that Mn may target sites other than zinc‐binding molecules, such as the Shank scaffolding proteins known to bind zinc in the PSD [84].

In our study, the lack of fluorescent markers for presynaptic compartments that are compatible with live cell imaging meant that Mn accumulation at the presynaptic level could not be studied. However, this accumulation is indirectly suggested by the detrimental effects of Mn on presynaptic mechanisms [83, 85]. Additional limitations may apply to our study. In adherent 2D cultures, organelles and cell compartments can be identified due to their natural spatial separation. For example, the nucleus and the Golgi apparatus can be distinguished by their characteristic size, shape, and position. The Golgi apparatus is located asymmetrically on one side of the nucleus. However, as the analyzed thickness spans the entire cell, partial overlap with other cellular structures can occur. For the PSD, superposition with other structures is not likely because this compartment is very thin (<50 nm). Another limitation of this study is the use of hippocampal neurons rather than basal ganglia neurons, which are the primary site of Mn accumulation. Primary cultures of globus pallidus neurons are technically challenging to establish and maintain, limiting their routine use in mechanistic studies. However, hippocampal neurons provide a well‐characterized model to study fundamental mechanisms of neurotoxicity that are largely conserved across neuronal subtypes. Furthermore, Mn has been detected in the human hippocampus following occupational exposure to welding fumes [23], as well as in animal models of environmental exposure through ingestion [20, 21, 22], or inhalation [18]. Nevertheless, future studies using basal ganglia–derived neurons or in vivo models will be needed to investigate region‐specific effects. For quantitative analysis of single‐cells, the time of analysis and the synchrotron experimental session availability posed additional limitations. As a result, for each condition, only two out of the three prepared biological replicates were analyzed. To strengthen the reliability of our findings, quantitative results must be validated using a larger number of biological replicates.

Our results demonstrate the significant potential of combining SXRF element imaging and protein fluorescence microscopy to identify sites of subcellular metal distribution, which is essential for understanding the mechanisms of metal neurotoxicity [52]. Another significant advantage of single‐cell SXRF spectro‐imaging is the ability to describe and quantify metal distribution in various cell types in co‐culture. Using this capability, we quantified the element content in neurons cultured alone or in co‐culture with astrocytes, showing that the latter contained approximately 40% less Mn (Figure 7). This indicates that astrocyte presence reduces neuronal Mn uptake, contributing to neuroprotection. In the absence of astrocytes, we also observed that Mn accumulates excessively in the Golgi region, resulting in a broader and higher local distribution (Figures 3a and 4a; Supporting Information S9 and S10). This may indicate dysfunction in the storage capacity of the Golgi apparatus, as has been reported in PC12 cells exposed to excess Mn [65].

The neuroprotective role of astrocytes is also supported by higher potassium levels in neurons co‐cultured with astrocytes compared to neurons cultured alone (Figure 7). Potassium loss is a marker of cytotoxicity that can be mediated by over‐activated potassium and/or ionotropic glutamate receptor channels [86]. Furthermore, Mn quantification in the ‘co‐culture condition’ suggests that astrocytes absorb three times more Mn than neurons (Figure 7). The sequestration of Mn by astrocytes may indirectly limit its availability to neurons. Astrocytes account for approximately 80% of total Mn content in the brain under physiological conditions [36], making them the primary pool of Mn. It is often assumed that astrocytes can accumulate 10‐ to 50‐fold more Mn than neurons [34, 35], although exact intracellular concentrations have not yet been measured in either cell type under identical conditions.

While high levels of Mn internalized by astrocytes may limit exposure to neurons, this comes at the cost of astrocytes becoming the target of toxic effects. Overexposure to Mn has been shown to alter glutamatergic function mediated by astrocytes in the hippocampus of a young mouse model of Alzheimer's disease [87]. Exposure to Mn in mice induced astrocyte dysfunction in the hippocampus, disrupting the glutamine–glutamate–GABA (Gln–Glu–GABA) metabolic cycle and reducing GABA synthesis [18]. Mn exposure affects the activity of astrocytic glutamine synthetase and glutamate transporters, leading to impaired neurotransmitter cycling, excitotoxicity, and reactive astrocytes that can exacerbate neuroinflammation mediated by NF‐κB and oxidative stress [88].

Overall, our results suggest that the primary mechanism of neuroprotection is Mn storage in the Golgi apparatus, involving both neurons and astrocytes. However, once the Golgi apparatus' Mn storage capability is exceeded, it becomes a primary target of neurotoxicity. Mn can reach other critical intracellular regions, such as the postsynaptic compartments in neurons. The Golgi apparatus' ability to detoxify Mn may be impaired by excessive exposure to Mn or by mutations in Mn efflux proteins such as SLC30A10 [70].

Our study emphasizes the importance of early detection and intervention in neurological disorders related to exposure to Mn, particularly in occupational and environmental settings. The most effective way to avoid excessive exposure to Mn is to prevent exposure in the first place. Numerous studies have shown the neurotoxic effects of Mn in children and adults, and this has recently resulted in the WHO modifying its guideline on the recommended value of Mn in drinking water [89]. While the previous guideline of 400 µg/L for Mn in drinking water was discontinued in 2012, the WHO recommended value was set at 80 µg/L in 2022, taking into account recent research on Mn neurotoxicology. This is a significant achievement in protecting the global population against Mn toxicity. Our study also emphasizes the need for further research into new therapeutic approaches for Mn detoxification that could enhance Mn efflux through the Golgi apparatus pathway. Such therapeutic interventions are urgently needed for pathologies associated with SLC30A10 and SLC39A14 mutations.

## Author Contributions

I.K. performed sample preparation, synchrotron experiments, and data treatment. A.K. performed synchrotron experiments and data treatment. L.C.C.H. provided assistance during synchrotron experiments and data treatment. H.C. and M.S. developed the new ID21 setup, and provided assistance during synchrotron experiments and data analysis. S.R. and P.B. developed the protocol, made measurements, and performed data treatment of the neurons viability assay. M.S. conceived the PSD95 fluorescent marker. N.P. and M.F.M designed cryo‐FLM experiments and provided assistance. D.C. conceived the experiments. R.O. and A.C. conceived the experiments, performed synchrotron experiments, interpreted the results, and wrote the manuscript. All authors edited the final manuscript and approved the final version.

## Conflicts of Interest

The authors declare no conflicts of interest.

## Supporting information




**Supporting File**: advs75095‐sup‐0001‐SuppMat.docx.

## Data Availability

The data that support the findings of this study are available from the corresponding author upon reasonable request.
