# Supplementary material for: Synchrotron XRF Imaging Reveals Manganese Accumulation in the Golgi and Post‐Synapses of Neurons and Enhanced Uptake in Astrocytes
Source: Adv Sci (Weinh). 2026 Apr 9;13(34):e20364. doi: 10.1002/advs.202520364 (PMC13285117; doi:10.1002/advs.202520364)
Supplement: Supplementary file 1 — Supporting File: advs75095‐sup‐0001‐SuppMat.docx. [file ADVS-13-e20364-s001.docx]

**Supporting Information**

**Synchrotron XRF Imaging Reveals Manganese Accumulation in the Golgi and Post-synapses of Neurons and Enhanced Uptake in Astrocytes**

Ines Kelkoul, Aiyarin Kittilukkana, Luis C.C. Huarte, Hiram Castillo-Michel, Murielle Salome, Stéphane Roudeau, Pauline Belzanne, Matthieu Sainlos, Noémie Pied, Monica Fernandez-Monreal, Daniel Choquet, Richard Ortega and Asuncion Carmona

**Supporting information S1:** illustration of the correlative procedure using ICY with eC-CLEM plugin.

**Supporting information S2:** elemental content in neurons, co-cultured neurons and astrocytes, expressed in ng/mm^2^.

**Supporting information S3a:** elemental concentrations in neurons, co-cultured neurons and astrocytes, expressed in mM expressed in mM.

The [cell thickness] was measured in living conditions, hydrated cells, and MW is the molecular weight of the element of interest.

$$C_{vol} \left[ mM \right]= \frac{C_{surf} \left[ ng/{mm}^{2} \right]}{cell thickness \left[ mm \right]\cdot MW [g/mol]}$$


**Supporting information S3b:** elemental concentrations expressed in mM, after removing the outliers.

**Supporting information S4**: measured thickness of neurons and astrocytes in living conditions (µm).

**Supporting information S5:** statistics from supplementary material 3b, expressed in mM and removing outliers, for phosphorous.

**Supporting information S5:** statistics from supplementary material 3, expressed in mM and removing outliers, for sulfur.

**Supporting information S5:** statistics from supplementary material 3, expressed in mM and removing outliers, for potassium.

**Supporting information S5:** statistics from supplementary material 3, expressed in mM and removing outliers, for calcium.

**Supporting information S5:** statistics from supplementary material 3, expressed in mM and removing outliers, for manganese.

**Supporting information S6:** SXRF imaging in neurons and astrocytes not exposed to manganese. a) Elemental distribution of P, S, K, Ca and Mn obtained by SXRF in single neuron, scan size 65 µm x 65 µm, step 0.5 µm/pixel, scan time 100 ms. b) Elemental distribution of P, S, K, Ca and Mn obtained by SXRF in single astrocytes, scan size 70 µm x 70 µm, step 0.5 µm/pixel, scan time 100 ms. Scale bars: 5 µm.

**Supporting information S7: manganese cytotoxicity assays.** Viability assay was performed in cultured primary neurons using the ReadyProbes™ Cell Viability Imaging Kit, Blue/Green. Manganese was added to the culture medium for 24h at different concentrations: 0-1-10-50-100-250-500-1000 µM. Normalized neuron viability, IC_10_ (inhibitory concentration 10%) and IC_50_ are shown. The plot presents three dose-response curves calculated by different models (Log-Logistic (LL4), Weibull Model 1 (W14), Weibull Model 2 (W24)) and the calculated weighted mean and the associated uncertainty (green curve). The viability was normalized to the control. The IC_10_ (249 µM) and IC_50_ (502 µM) values were calculated from the mean curve. n, is the total number of cells analyzed (131,472).


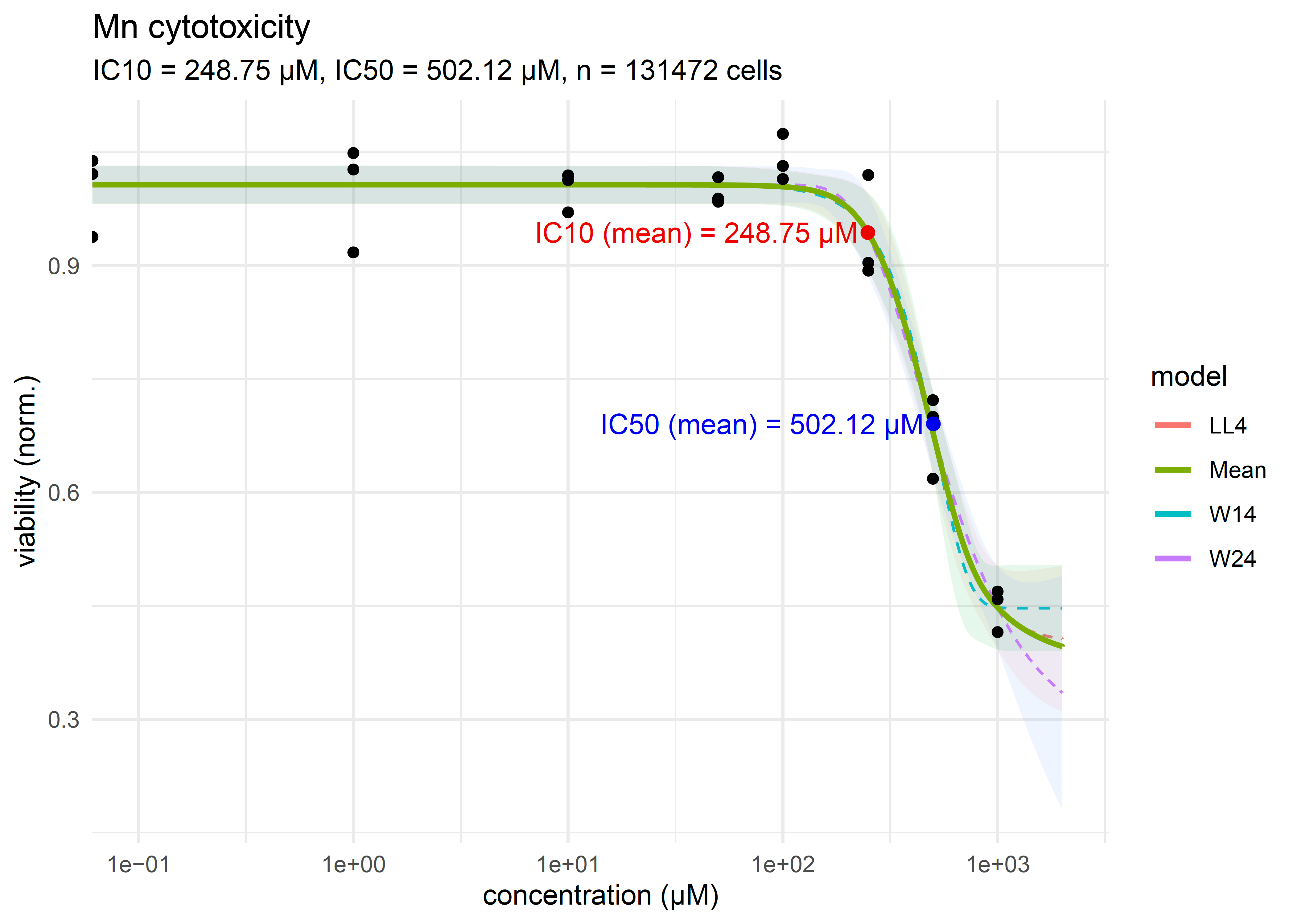


**Supporting information S8:** SXRF imaging in astrocytes exposed to manganese

**Supporting information S9:** SXRF imaging in neurons exposed to manganese in the presence of astrocytes.

**Supporting information S10:** SXRF imaging in neurons exposed to manganese in the absence of astrocytes.

**Supporting information S11:** SXRF imaging in the dendritic network of neurons exposed to manganese. a) Elemental distribution of P, K, Ca and Mn obtained by SXRF, scan size 45 µm x 60 µm, step 0.15 µm/pixel, scan time 50 ms. b) Merged images of Mn and K evidencing Mn in the neuronal branching, and images of Mn and Ca showing colocalization. c) zoomed region (white square in b) showing Mn along dendrites and Mn and Ca colocalization.

**Supporting information S12.** Elemental imaging in the dendritic spines of primary neurons exposed to Mn. a) Confocal fluorescence images of tubulin (magenta) and PSD-95 (green) in living primary hippocampal neurons at DIV21. b) Cryo-FLM images of the framed region in a), showing fluorescence of tubulin (magenta) and PSD-95 (green). c) Elemental distribution of Zn and Mn obtained by SXRF in the same region as b), scan size 50 µm x 75 µm, step 0.2 µm/pixel scan time 50 ms/pixel. d) Superimposed images of Mn (red), PSD-95 (green) and tubulin (magenta) and superimposition of Zn (red), PSD-95 (green) and tubulin (magenta). Zoom in the regions framed in d) to show e) Mn and Zn reaching the post synaptic compartments (blue squares), f) Mn in the close vicinity of dendritic spines (green squares) and g) Mn along the microtubules (red squares). Scale bar 10 µm for panel a; 2 µm for panels b, c and d; 500 nm for panels e, f and g.
